# 复合共价有机框架材料用于食品中新污染物分析的样品前处理技术研究进展

**DOI:** 10.3724/SP.J.1123.2025.08002

**Published:** 2026-07-08

**Authors:** Zeyi SUN, Guangnian YUAN, Yuanye NIU, Jiping MA

**Affiliations:** 青岛理工大学环境与市政工程学院，山东 青岛 266520; School of Environmental and Municipal Engineering，Qingdao University of Technology，Qingdao 266520，China

**Keywords:** 复合共价有机框架, 食品样品, 样品前处理技术, 新污染物, composite covalent organic framework materials, food samples, sample pretreatment techniques, new pollutants

## Abstract

新污染物是指新近发现或被关注、对生态环境或人体健康存在风险、尚未纳入管理或现有管理措施不足以有效防控其风险的化学物质，具有生物毒性、环境持久性和生物累积性等特征，对人类健康构成潜在威胁。近年来，食品中新污染物的检出频率增加，亟须开发高效的食品新污染物检测技术。目前，用于食品中新污染物分析的前处理技术多种多样，而其核心要点在于吸附材料的制备及选择。共价有机框架（COF）是由轻元素通过共价键连接形成的多孔晶体材料，具有高度有序的晶体结构、可调控的孔径、可功能化的表面特性，以及优异的化学稳定性和热稳定性。复合COF材料是将COF与其他材料通过物理或化学方法结合，形成具有协同效应的新型复合材料，兼具COF与其他材料的独特性能。本文综述了食品新污染物分析中常用的样品前处理技术，包括固相萃取（SPE）、固相微萃取（SPME）、搅拌棒吸附萃取（SBSE）、分散固相萃取（DSPE）和磁固相萃取（MSPE）等。本文详细介绍了复合COF材料的种类，包括磁性COF、海绵-COF、分子印迹聚合物-COF、金属有机框架-COF、静电纺丝-COF及其在食品新污染物分析样品前处理中作为吸附材料的应用，同时对复合COF材料在食品样品前处理领域的未来发展进行了展望。

## Abstract (en)

New pollutants are recently identified or recognized chemical substances. They pose risks to ecosystems or human health. Many are not yet regulated or lack effective control measures. These pollutants show biological toxicity， environmental persistence， and bioaccumulation. They threaten human health. In recent years， their detection frequency in food has increased. Efficient detection technologies are urgently needed. Sample pretreatment is key for analyzing new pollutants in food. The core of pretreatment lies in the preparation and selection of adsorbent materials. Covalent organic frameworks （COFs） are porous crystalline materials. They are formed by light elements linked through covalent bonds. COFs have highly ordered crystal structures. Their pore sizes can be adjusted. Surface properties are functionalizable. They show excellent chemical and thermal stability. Composite covalent organic framework materials combine COFs with other materials. This is achieved through physical or chemical methods. The composites exhibit synergistic effects. They retain the unique properties of both COFs and the other materials. This article reviews common sample pretreatment techniques for new pollutants in food. These include solid-phase extraction （SPE）， solid-phase microextraction （SPME）， stir bar sorptive extraction （SBSE）， dispersive solid-phase extraction （DSPE）， and magnetic solid-phase extraction （MSPE）. SPE is a chromatographic technique. It removes impurities from solid or liquid samples. It also enriches target compounds. SPE offers high enrichment factors and low solvent use. It is easy to automate. SPME balances samples between solid and liquid phases. It integrates sampling， extraction， and concentration. SPME uses little or no solvent. It is simple and automatable. It can be coupled with other techniques online. SBSE evolved from SPME. It has a larger stationary phase volume and higher capacity. SBSE uses a stir bar with a magnetic core. The bar is coated with an extraction layer. Stirring ensures full contact with analytes. SBSE is solvent-free or uses minimal solvent. It is accurate， fast， and easy to automate. DSPE disperses adsorbents into sample matrices. It increases contact area between adsorbents and analytes. DSPE simplifies sample processing. It avoids sample loss. MSPE uses magnetic or magnetizable materials as adsorbents. It captures target analytes efficiently. MSPE is simple to prepare and separate. Pipette-tip-SPE （PT-SPE） is a newer technique. It packs adsorbents into pipette tips. PT-SPE is flexible， low-cost， and needs small sample volumes. This article details types of composite COF materials. These include magnetic COF （MCOF）， sponge-COF， molecularly imprinted polymer-COF （MIP-COF）， metal-organic framework-COF （MOF-COF）， and electrospun-COF. MCOF combines COFs with magnetic nanoparticles. It enables quick separation under a magnetic field. Sponge-COF grows COFs on sponge fibers. It enhances adsorption capacity and mass transfer. MIP-COF integrates molecularly imprinted polymers with COFs. It offers specific recognition sites. MOF-COF combines metal-organic frameworks with COFs. It introduces metal active sites. Electrospun-COF embeds COFs into polymer nanofibers. It improves mechanical performance and stability. These composite materials are utilized in the pretreatment of food samples， where they effectively enrich trace levels of new pollutants， including perfluoroalkyl substances， antibiotics， personal care products， endocrine disruptors， and flame retardants， within complex food matrices. When coupled with analytical techniques such as high-performance liquid chromatography （HPLC）， HPLC-tandem mass spectrometry （HPLC-MS/MS）， gas chromatography （GC）， gas chromatography-mass spectrometry （GC-MS）， molecular fluorescence spectroscopy， and Raman spectroscopy， they facilitate highly accurate and sensitive detection of these new pollutants in food products. Future directions include improving synthesis methods. Current methods are time-consuming and costly. New techniques like water-phase synthesis are promising. Multifunctional composites are needed. They should adsorb multiple pollutant types. Automated and high-throughput extraction technologies will be developed. Green and scalable production processes are essential for industrial applications. In conclusion， composite COF materials show great potential. They enhance the efficiency and accuracy of food pollutant analysis. Further research will expand their applications and improve performance.

近年来，食品安全问题已成为全球关注的焦点。污染物引发的食品安全隐患普遍存在，长期影响食品的安全供应。新污染物是指具有生物毒性、环境持久性、生物累积性等特征的有毒有害化学物质。目前，国际上广泛关注的新污染物有全氟化合物、抗生素、个人护理品、内分泌干扰物、阻燃剂等。这些新污染物主要通过畜牧业和水产养殖业使用、食品包装材料释放、环境介质迁移以及食品生产加工等途径进入食品中^［1］^。新污染物通过多种途径进入食品后，会对人类健康造成多重危害，引发免疫系统受损、发育障碍、慢性疾病（如肥胖、糖尿病）、生殖健康问题以及对心血管系统的潜在危害^［2］^。此外，新污染物还可能通过食物链的生物累积和生物放大作用，进一步造成对人类健康的负面影响^［3］^。近年来，我国已对食品及食品接触材料中新污染物的限值标准做出明确规定，对食品接触材料中全氟辛酸和全氟辛烷磺酸的含量分别限制在0.025 mg/m²和0.01 mg/m²以下^［4］^，564种农药在不同种食品中的最大残留范围为1~50 000 ng/g^［5］^，对动物源性食品中104种抗生素的最大残留限量范围为0.3~12 000 ng/g^［6］^。随着各类化学物质的大量生产和使用，新污染物在食品中的检出频率不断增加^［7］^。

食品中新污染物分析的仪器分析方法主要包括高效液相色谱法（HPLC）^［8］^、高效液相色谱-串联质谱法（HPLC-MS/MS）^［9］^、气相色谱法（GC）^［10］^和气相色谱-质谱法（GC-MS）^［11］^、分子荧光光谱法^［12］^、拉曼光谱法^［13］^等。食品样品通常成分复杂，新污染物的含量较低，并且含有多种干扰物质，直接对食品样品进行分析往往难以获得准确的结果。前处理技术可以有效地去除食品样品中的杂质和干扰物，提高分析的准确性和重现性，确保分析结果的可靠性。目前，用于食品中新污染物分析的多种前处理方法通常涉及多个复杂步骤，但这些方法的核心要点在于吸附材料的制备及选择。

共价有机框架（COFs）由Yaghi等^［14］^于2005年首次报道，是一种由轻元素（如氢、硼、碳、氮和氧）组成，通过共价键连接形成的多孔晶体材料。COF材料完全由强共价键连接而成，因此展现出较高的热稳定性，同时具有极大的比表面积，在多孔材料领域具有显著优势^［15］^。此外，COF具有孔状结构可调控、易于修饰等优点，在吸附方面展现出较大的应用潜力^［16］^。COF还能够通过引入特定的官能团或化学基团，与目标分子之间形成氢键、静电相互作用或*π-π*堆积等特异性化学作用，有利于增强COF材料的吸附能力和选择性^［17］^。复合COF材料是指以COF材料为基础，与海绵、分子印迹聚合物（MIP）、金属有机框架（MOF）、静电纺丝等其他材料复合而成的新型材料。复合COF结合了COF的多孔性和其他材料的特性，如增加吸附材料对目标化合物的选择性^［18］^、使材料易于与水相分离、具有更高的机械强度^［19］^等。这些特性使得COF材料在固相萃取（SPE）、固相微萃取（SPME）、搅拌棒吸附萃取（SBSE）、磁固相萃取（MSPE）和分散固相萃取（DSPE）等食品样品前处理技术中发挥着重要作用^［20］^。

目前，已有一些综述文章针对COF及复合COF材料在食品样品前处理中的应用进行了总结。Xin等^［21］^综述了COF吸附剂在食品污染物（包括多环芳烃、生物胺、杀虫剂、重金属离子、非法添加剂、生物毒素等）富集分析中的应用，但未关注食品中新污染物的检测。Fu等^［22］^重点介绍了近年来磁性COF材料在食品污染物的富集和分析中的应用进展，但未介绍其他类型复合COF材料的应用。基于此，本文系统总结了用于食品中新污染物样品前处理的复合COF材料类型，包括磁性共价有机框架（MCOF）、海绵-共价有机框架、分子印迹聚合物-共价有机框架（MIP-COF）、金属有机框架-共价有机框架（MOF-COF）以及静电纺丝-共价有机框架，综述了食品新污染物分析领域常用的样品前处理方法，包括SPE、SPME、SBSE、DSPE、MSPE，以及新兴的针尖固相萃取（PT-SPE）的应用进展，并涵盖了近5年来复合COF材料在食品新污染物分析检测中的研究应用（见图1），旨在为复合COF材料在食品样品前处理的应用提供一定参考，以期为食品新污染物检测领域提供新的视角和研究方向。

**图1:**
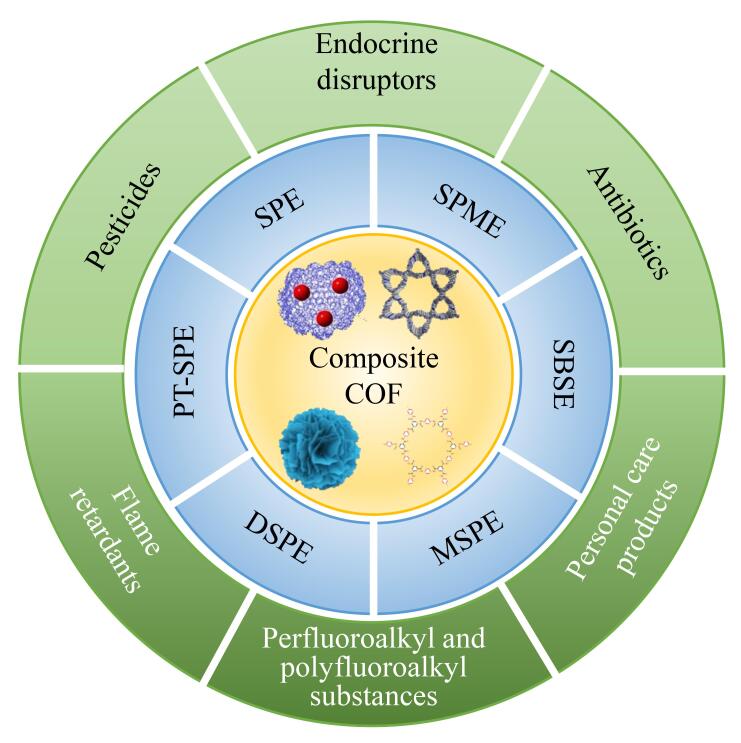
复合COF材料在食品样品前处理中的应用

## 1 食品中新污染物分析的样品前处理技术

SPE是一种基于色谱技术发展而来的样品前处理方法^［23］^，主要用于去除固体和液体样品提取液中的杂质，或者富集浓缩目标化合物，具有富集倍数高、有机溶剂用量少、易于自动化操作等优势。SPME是基于样品在固-液两相间的平衡而建立起来的集进样、萃取、浓缩功能于一体的技术。SPME技术具有不用或少用溶剂、操作简便、易于自动化和能够与其他技术在线联用等优点，与其他常用的富集技术相比，克服了传统的液液萃取法需使用大量溶剂和样品、处理时间长、操作步骤多的缺点^［24］^。SBSE源于SPME，与SPME相比具有更高的固定相体积、萃取容量和萃取回收率。SBSE技术利用内置磁芯的搅拌棒，在其表面涂覆一层萃取涂层，通过搅拌使涂层与分析物充分接触并吸附目标化合物^［25］^。SBSE技术不仅操作简单，而且能够实现样品的大容量萃取和高灵敏度分析，具有无溶剂或少溶剂、自动化程度高、准确度高、快速简便等优点。DSPE是一种将吸附剂分散到样品基质或其提取物中进行萃取的食品样品前处理技术，能够提高吸附剂颗粒与被分析物之间的接触面积，更有效地吸附和分离目标分析物^［26］^。DSPE能够简化样品处理的流程，避免样品损失，从而成为近年来食品样品前处理技术的热点研究方向之一^［27］^。MSPE是一种新型的分散固相萃取技术，利用磁性或可磁化材料作为吸附剂来捕获目标分析物。MSPE技术的关键在于磁性吸附剂的选择和制备^［28］^。与传统前处理技术相比，MSPE具有制备简便、易于分离等优点^［29］^。近年来，用于食品样品前处理的技术还有针尖PT-SPE，其通过将少量吸附剂装入移液枪头中，用于食品样品的高效萃取或纯化过程。PT-SPE的操作简便，成本低廉，应用更加灵活，只需要较少的样品体积^［30］^。

## 2 复合COF材料

复合COF材料是将COF与其他材料通过物理或化学方法结合形成的具有协同效应的新型复合材料。在食品样品前处理领域，复合COF材料的主要类型包括磁性共价有机框架、海绵-共价有机框架、分子印迹聚合物-共价有机框架、金属有机框架-共价有机框架以及静电纺丝-共价有机框架等。它们既结合了COF材料多孔性、传质快和可设计等特点，又有其他材料的独特性能，从而在食品新污染物分析领域表现出了良好的应用效果。

### 2.1 磁性共价有机框架复合材料

对MSPE技术而言，制备具有高效吸附能力的磁性材料，将其用于食品样品中目标化合物的检测至关重要。但单一的磁性纳米粒子容易出现团聚现象，且表面官能团较为单一，难以获得满意的萃取效果。因此，对磁性材料进行功能化修饰，提升其富集能力或选择性成为目前研究的热点^［31］^。而COF表面的官能团能够为磁性材料的功能化修饰提供相关的活性位点，使构建的MCOF具有特定的功能基团，增强对目标化合物的选择性吸附^［32］^。此外，MCOF的超顺磁性使其能够在外部磁场的作用下快速分离，无需特殊的仪器或设备，极大简化了样品前处理流程^［33］^。Li等^［34］^利用溴化乙锭（EB）和1，3，5-三甲酰基间苯三酚（Tp）作为结构单元，通过溶剂热反应合成了磁性复合共价有机框架材料EB-COF@Fe_3_O_4_，并将其用作MSPE吸附剂，结合HPLC检测，开发了一种用于果汁和番茄样品中5种苯甲脲类杀虫剂（BUs）的灵敏测定新方法（见图2）。

**图2:**
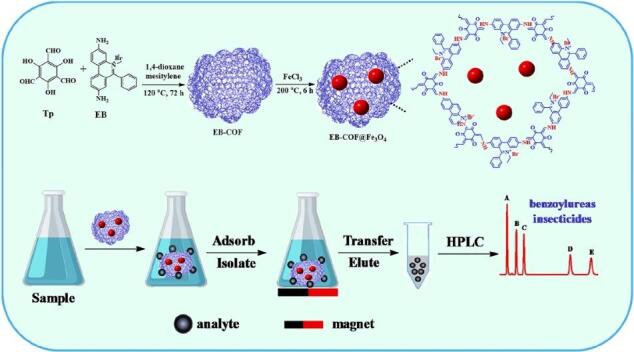
EB-COF@Fe_3_O_4_的制备及MSPE流程图^［34］^

### 2.2 海绵-共价有机框架复合材料

海绵是一种轻质、高弹性和具有良好机械性能的材料，通过不同的合成策略，如反应接种^［35］^或熔融聚合^［36］^，可以实现COF在海绵纤维上的均匀生长，从而形成海绵-共价有机框架复合材料。这种复合材料不仅具有COF的高比表面积和选择性吸附特性，还能够利用海绵的三维多孔结构来增强材料的吸附容量和传质效率。Li等^［37］^以三聚氰胺泡沫（MF）为载体，*N，N，N′，N′*-四（4-氨基苯基）-1，4-苯二胺（TPDA）和2，2′-联吡啶-5，5′-二甲醛（Bpy）为配体，通过直接原位生长的方式，一锅合成了异孔MF@COF复合材料（见图3a）。该方法采用乙腈涡旋提取，离心后加入MF@COF，再涡旋30 s即可有效去除花生、油菜籽、核桃、芝麻、大豆和玉米中的脂肪基质（甘油三酯和游离脂肪酸）。MF@COF与油相中的甘油三酯和游离脂肪酸相互作用，实现脂肪基质的优先吸附，以防止脂肪积累在色谱柱中，影响分析的灵敏度和仪器的使用寿命，同时实现了氨基甲酸酯、有机磷、新烟碱类农药等新污染物的完全回收（见图3b）。

**图3:**
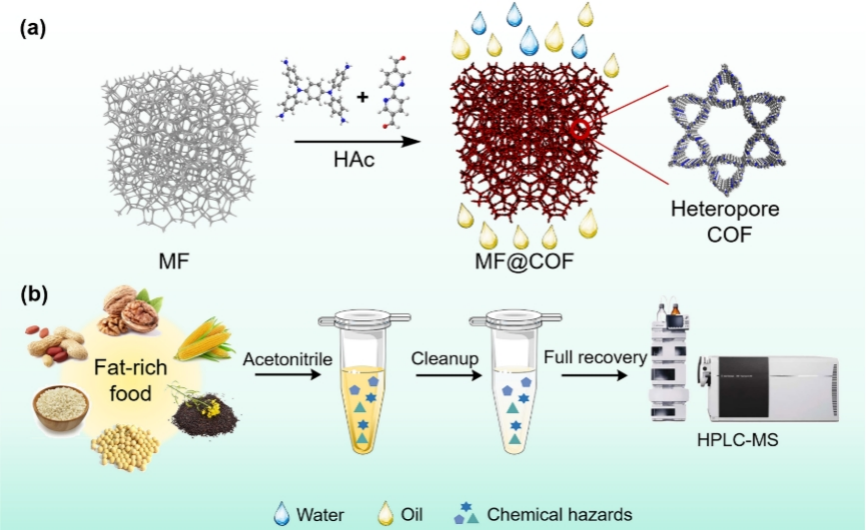
（a）疏水MF@COF复合材料的制备和（b）MF@COF在富含脂肪样品处理中的应用^［37］^

### 2.3 分子印迹聚合物-共价有机框架复合材料

MIP-COF是一种结合了MIP的识别特性和COF的多孔性质的复合型功能材料。MIP是一种通过分子印迹技术（MIT）制备的具有特异性识别位点的合成聚合物，具有结构可预测性、识别特异性和应用普适性等独特优势，在样品预处理、色谱分离、化学和生物传感等领域得到了广泛应用^［38］^。MIP材料所具有的特定智能识别位点，使其能够对目标分子进行高选择性识别和结合^［39］^。相比天然识别材料（如抗体），MIP展现出高效的结合能力，同时具备制备流程简便、成本低廉、稳定性卓越和重复利用率高等优势。MIP-COF结合了二者的特性和优点，不仅能够对特定目标分析物进行选择性识别，还能够利用COF的多孔结构实现高效的样品富集和分离。Su等^［40］^采用多巴胺作为功能单体和交联剂，以恩诺沙星为模板分子，通过自聚合反应，在具有不可逆二噁英键连接的COF表面生成印迹腔体，制备出一种MIP-COF用作吸附剂，结合HPLC进行检测，能够实现鸡肉、鱼肉、虾肉和猪肉样品中恩诺沙星、诺氟沙星和环丙沙星3种氟喹诺酮类抗生素（FQs）的同时测定（见图4）。

**图4:**
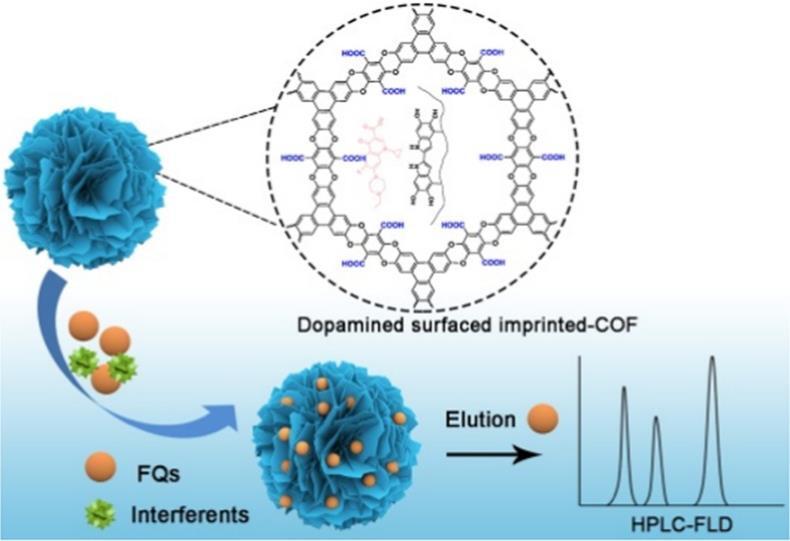
MIP-COF的结构及其应用^［40］^

### 2.4 金属有机框架-共价有机框架复合材料

MOF是由有机配体和金属簇通过配位键构建的多孔功能材料，具有丰富的官能团，可以通过*π-π*共轭、氢键和疏水相互作用力捕获污染物^［41］^，具有比表面积大、孔径易于调节、活性位点分布均匀等特点^［42］^。MOF-COF复合材料通过将金属配合物与有机连接体结合起来，既保留了COF的高化学稳定性，又引入了MOF的金属活性位点，能够提高材料的吸附和分离性能，同时拓展了材料的功能性和应用潜力^［43］^。Azadkish等^［44］^合成了Mn-TA/COF纳米复合材料，通过溶剂热法将其应用于SPME纤维中，对农业用水、洋葱、莴苣和葡萄样品中的农药氟乐灵进行富集萃取和超痕量分析。

### 2.5 静电纺丝-共价有机框架复合材料

静电纺丝技术是一种利用高电压使聚合物溶液或熔体在电场作用下形成射流，随着溶剂挥发或冷却固化，拉伸成直径从纳米到微米级别超细纤维的加工方法。静电纺丝技术简单、成本效益高且用途广泛^［45］^。静电纺丝-共价有机框架复合材料是通过静电纺丝技术将COF嵌入到聚合物纳米纤维中制备而成的一种高性能复合材料，结合了静电纺丝纳米纤维的优异力学性能和COF材料的结构可调性，展现出良好的稳定性、高效的吸附能力以及可重复使用性^［46，47］^。Wang等^［48］^以对苯二胺、均苯三甲酰氯和聚丙烯腈（PAN）为单体，采用静电纺丝法制备了PAN@COF-SCU1纳米纤维，将其置于0.2 mL移液枪头中。取均质化的鸭肉和草鱼样品，加入甲酸-乙腈-水混合溶液提取，经过超声、离心、浓缩、复溶后，通过装有PAN@COF-SCU1纳米纤维的移液枪头，氨水-甲醇溶液洗脱后，采用HPLC分析样品中的土霉素、四环素和氯四环素（见图5）。

**图5:**
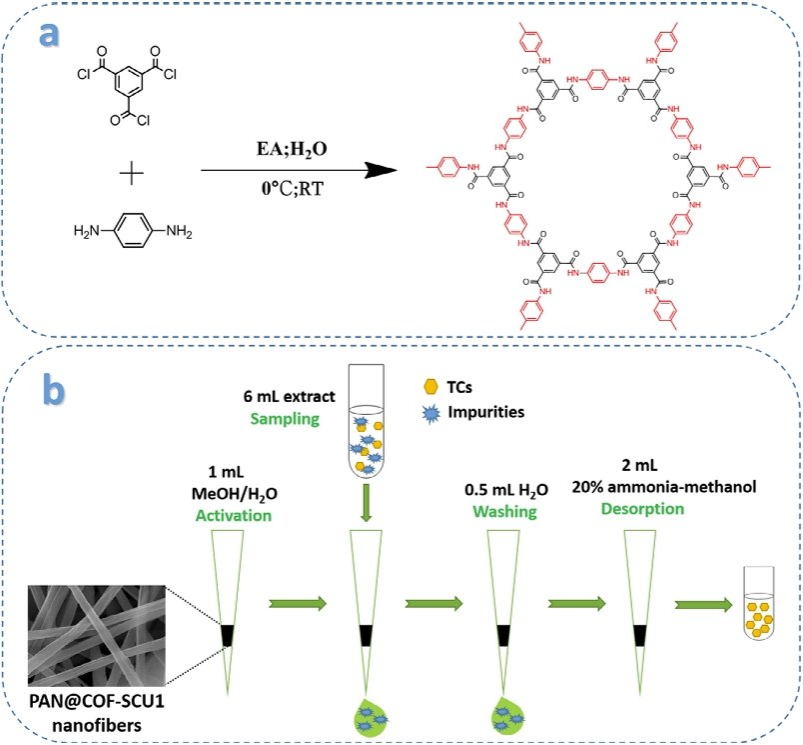
（a）COF-SCU1制备和（b）提取四环素类抗生素的PT-SPE程序示意图^［48］^

## 3 COF材料在食品新污染物分析样品前处理中的应用

食品污染物可能来源于环境污染、生产加工过程中的化学反应、食品包装材料的迁移物，或是由于食品掺假、造假等原因混入食品中。随着现代经济的快速发展，食品安全问题日益凸显，食品中新污染物分析检测的需求日益增加。常见的食品新污染物包括全氟化合物、抗生素、个人护理品、内分泌干扰物、阻燃剂、农药等。

### 3.1 全氟化合物

全氟化合物（PFAS）是一类持久性有机污染物（POPs），其分子结构中含有全氟烷基或全氟烷氧基基团，具有极强的化学稳定性和热稳定性。PFAS因其持久性、生物累积性和毒性，对环境和人类健康构成了潜在危害，可能产生致癌性、生殖毒性、免疫系统和内分泌系统紊乱等不良问题^［49］^。Han等^［50］^通过赫克交叉偶联反应，在溴化共价有机框架（Br-COF）中引入4-乙烯基吡啶作为配体，合成了含有吡啶（Py）基团的吡啶功能化共价有机框架（Py-COF）。样品粉碎后置于离心管中，加入甲醇溶解，涡旋、离心取出上清液，过滤稀释。将Py-COF作为涂层的SPME纤维浸泡于处理得到的样品上清液中，富集其中的全氟聚醚羧酸（PFECAs），结合HPLC-MS/MS测定马铃薯、生菜、黄瓜、梨、橘子、香蕉、鸡肉、牛肉、带鱼中的PFECAs。该方法对于不同样品中PFECAs的检出限为0.001~0.004 ng/g，定量限为0.003~0.012 ng/g。

### 3.2 抗生素

抗生素类药物具有抑制或杀灭微生物的作用，被广泛用于治疗人类和其他动物细菌感染等疾病，以及用作畜禽畜牧业的饲料添加剂。抗生素主要分为*β*-内酰胺类（如青霉素类、头孢菌素类、碳青霉烯类等）、四环素类、氯霉素类、喹诺酮类、磺胺类、大环内酯类、林可霉素类等多种类型，每类抗生素都有其独特的抗菌谱和作用机制。然而，随食品摄入过量抗生素不仅会直接导致人体内微生物群落失调，增加耐药菌的定植和传播风险，还可能通过环境排放进一步加剧抗微生物耐药性的扩散，对人类健康和生态系统造成更广泛的潜在危害^［51，52］^。Wang等^［53］^制备了磺酸功能化的共价有机框架（COF-SO_3_）作为SBSE的涂层，将牛奶样品用纯净水稀释5倍后，用于从牛奶样品中富集恩诺沙星、洛美沙星、斯帕沙星，结合HPLC技术，建立了牛奶样品中3种FQs的SBSE-HPLC检测方法。该方法避免了牛奶样品传统前处理方法中蛋白质沉淀、脱脂等复杂步骤，对于3种抗生素药物的检出限较低，为1.20~2.62 ng/mL，定量限为4.00~8.75 ng/mL，适用于牛奶中3种痕量抗生素的分析检测。Chen等^［42］^将COF修饰在磁性MOF表面，得到新型MOF@COF杂化磁性纳米球作为吸附剂，分散在经切碎、离心、浓缩、溶解与过滤处理后的肉类样品乙腈提取液中，再取50 μL提取液用去离子水稀释至20 mL，用于后续的MSPE，富集猪肉、鸡肉和虾肉样品中的6种磺酰胺类抗生素（SAs），并采用乙腈解吸，解吸液经磁分离和过滤后注入HPLC进行分析。该方法的提取时间和解吸时间仅为5 min和2 min，大大提高了分析效率，且操作简便，6种SAs的检出限为0.1~0.5 ng/mL。Wang等^［54］^通过超声波原位生长法，以TPDA和（1，1′-联苯）-4，4′-二羧醛（BPDA）为配体，形成了具有异孔结构的COF材料，将其负载在聚多巴胺（PDA）包裹的海绵材料上，并通过聚二甲基硅氧烷（PDMS）包覆，合成了PDMS@COF@PDA海绵材料。这种COF材料与甘油三酯中的脂肪酸链之间存在疏水相互作用，且材料表面的极性基团能够与甘油三酯中的羟基形成氢键，从而高效去除样品中的甘油三酯。该方法采用简单的涡旋处理就能够从猪油和猪肉样品中去除超过98%的干扰基质成分甘油三酯，并减弱COF与抗生素之间的疏水性和*π-π*相互作用，实现样品中44种具有不同理化性质抗生素的萃取和HPLC-MS检测。

### 3.3 个人护理品

个人护理品（PCPs）是指在日常生活中用于个人卫生、美容和护理的各种产品，常见的种类包括紫外线吸收剂（如二苯甲酮类）、防腐剂（如对羟基苯甲酸酯类）、抗菌剂（如三氯生）及合成麝香等。个人护理品中的部分有机物成分常被用于食品包装材料的生产过程，并可能在食品储存或加工过程中迁移到食品中。长期接触此类成分会引发皮肤刺激、内分泌紊乱和癌症，甚至对人类生殖系统产生负面影响^［55］^。Phalipat等^［56］^以1，3，5-三甲醛苯和对苯二胺为原料，通过席夫碱反应合成了亚氨基COF，并与海藻酸钙水凝胶结合，制备了一种复合COF/水凝胶吸附剂，用于涡流辅助DSPE，其能够通过疏水作用、*π-π*和氢键相互作用有效吸附蛋黄酱、婴儿奶粉和咖啡奶精样品中的3种对羟基苯甲酸酯和2种合成酚类抗氧化剂。样品需先加入乙腈沉淀蛋白质和脂肪，离心后蒸干上清液，加入去离子水制成样品溶液，再将COF/水凝胶吸附剂加入含有样品溶液的试管中，涡旋混合进行吸附，采用乙腈洗脱，并将洗脱后的分析物过滤后注入HPLC进行分析。该方法的检出限为0.25~0.50 ng/g，定量限为1.00~2.00 ng/g，适用于蛋黄酱、婴儿奶粉和咖啡奶精中对羟基苯甲酸酯和合成酚类抗氧化剂的分析检测。

### 3.4 内分泌干扰物

内分泌干扰物（EDCs）是一种可以模拟或干扰生物的内源激素，并与激素受体结合或抑制正常生物反应的化学物质，即使生物体内存在微量EDCs，也会破坏生物体的正常功能，导致其生长发育受损^［57］^。常见的EDCs包括雌激素、双酚A和烷基酚类化合物等。Liu等^［58］^以Fe_3_O_4_纳米颗粒为磁芯，硼酸（BA）功能化COF为外壳层，通过后修饰合成了硼酸功能化核壳结构Fe_3_O_4_@COF@BA，将制备的复合材料作为MSPE吸附剂，结合HPLC-MS/MS，富集并检测了牛肉、鸡肉和猪肉样品中的双酚A、己烯雌酚、六雌酚、二烯雌酚、辛基酚、壬基酚。该方法检出限低至0.08~0.72 ng/g，可用于牛肉、鸡肉和猪肉样品中6种EDCs的提取和检测。

### 3.5 阻燃剂

阻燃剂是一类能够减缓或阻止可燃材料燃烧的化学物质。近年来，阻燃剂类物质被广泛应用于塑料、树脂、电线电缆涂层、电子产品和纺织品等生产与生活用品材料中^［59，60］^。虽然阻燃剂在防火安全方面发挥了重要作用，但部分阻燃剂（如溴化阻燃剂）会在食品包装或加工过程中释放并迁移到食品中^［61］^，对人体健康产生多种负面影响，包括但不限于神经毒性、心脏毒性、生殖和发育毒性、细胞毒性和内分泌干扰效应^［62］^。Gao等^［63］^以Tp和联苯胺（BD）等为原料合成了一种TpBD-COF材料，将其涂覆在SPME石英纤维表面后，浸入1 mL橙汁样品中搅拌提取，用于吸附和富集橙汁中的四溴双酚A（TBBPA），结合恒流解吸电离质谱技术，建立了一种快速检测橙汁中痕量TBBPA的方法。该方法具有良好的灵敏度和稳定性，橙汁样品中TBBPA的检出限和定量限分别为0.92 ng/mL和3.1 ng/mL，可在7 min内完成检测。Liu等^［64］^以1，3，5-三（4-氨基苯基）三嗪（TAPT）和2，5-二甲氧基-1，4-二苯甲醛（DMTA）为单体，采用溶剂热法合成了新型DSPE吸附剂TAPT-DMTA-COF，用于吸附牛奶和鱼肉中的多溴联苯醚（PBDEs）。对于两种样品的前处理和分析，需要先将牛奶样品均质化、脱脂，将鱼肉样品剪碎、均质化并制备成鱼肉浆液，再将吸附剂加入含有样品溶液的离心管中，以1，2-二氯苯-正丁醇-乙酸混合溶液为提取液，经振荡、离心后，使用正己烷洗脱吸附剂中的PBDEs，并在去除样品溶液中的正己烷后，注入气相色谱-串联质谱联用仪（GC-MS/MS）进行检测。该方法对于牛奶和鱼肉样品中5种PBDEs的检出限为0.03~0.13 ng/mL，定量限为0.10~0.43 ng/mL，并显示出良好的回收率。

### 3.6 农药

农药包括杀虫剂、除草剂、杀菌剂和植物生长调节剂等，其过度使用会污染环境和农产品。农药暴露可能导致神经损伤、遗传毒性以及代谢紊乱等多种不良影响^［65］^。Li等^［66］^通过可控原位生长法制备了核壳型MCOF材料Fe_3_O_4_@COF作为MSPE吸附剂，检测水果和市售果汁中残留的5种苯并咪唑杀菌剂，其检出限和定量限范围分别为2.5~2.9 ng/mL和8.8~9.7 ng/mL。Du等^［67］^采用两步法合成了氨基修饰的COF（TpPa-NH_2_）@Fe_3_O_4_纳米复合材料，将其作为MSPE的吸附剂，结合荧光光谱法对白菜、苹果和蜂蜜样品中的氨基甲酸酯类农药西维因进行了检测，该方法检出限为0.012 ng/g。COF（TpPa-NH_2_）@Fe_3_O_4_能够通过*π-π*堆积和疏水作用，直接从样品中快速捕获西维因，并在5 min内达到最大吸附量。

## 4 结论及展望

COF材料作为多孔有机框架材料的典型代表，具备高度有序的结构、可调控的孔隙尺寸、优异的化学与热稳定性以及表面功能的可调节性，已被应用于多种食品分析的样品前处理中，能够有效提高目标分析物的萃取效率。目前，设计开发出的多种复合COF材料在富集食品样品中的新污染物方面展现出了优异的性能，有效提高了分析方法的灵敏度和准确性。

样品基质的复杂性、新污染物的低含量以及其他新污染物的出现等使食品样品前处理面临新的挑战，这一领域未来的发展方向包括：（1）目前复合COF材料的合成方法存在合成周期长、成本高、纯度低等问题。除了传统的溶剂热法、微波辅助法、离子热合成法、机械研磨法等，“外部助剂”辅助固相合成法、后合成改性等新的合成方法正逐步被更多研究采用。未来，需要进一步开发和优化新的合成方法，以提高复合COF材料的合成效率和纯度，降低成本。（2）开发具有多重功能的复合COF材料。通过在框架中引入不同官能团或结合选择性材料，使复合COF材料能够同时吸附多种类别的目标分析物。（3）发展基于复合COF材料的新型萃取技术，如在线、高通量、自动化等。（4）开发可规模化、低成本、环境友好的复合COF材料合成与加工工艺。当前，大多数复合COF材料的制备仍处于实验室阶段，步骤复杂、成本较高或需使用有毒溶剂。未来研究需要探索更简单、高效、绿色的合成路线（如水相合成、机械化学合成、连续流合成等），以及易于放大生产的制备方法，推动复合COF材料从实验室走向工业化生产和实际应用。
